# Altered gut microbiota and gut-derived p-cresyl sulfate serum levels in peritoneal dialysis patients

**DOI:** 10.3389/fcimb.2022.639624

**Published:** 2022-09-27

**Authors:** Manchen Bao, Pan Zhang, Shulan Guo, Jianzhou Zou, Jun Ji, Xiaoqiang Ding, Xiaofang Yu

**Affiliations:** ^1^ Department of Nephrology, Zhongshan Hospital, Fudan University, Shanghai, China; ^2^ Shanghai Institute of Kidney Disease and Dialysis, Shanghai, China; ^3^ Shanghai Key Laboratory of Kidney disease and Blood Purification, Shanghai, China; ^4^ Shanghai Medical Center of Kidney, Shanghai, China

**Keywords:** Uremic toxins, Peritoneal Dialysis, residual renal function (RRF), Gut micobiota, p-cresyl sulfate (PCS)

## Abstract

Peritoneal dialysis (PD) is a renal replacement therapy for end-stage renal disease. Gut microbiota-derived uremic solutes, indoxyl sulfate (IS), p-cresyl sulfate (PCS), and trimethylamine-N-oxide (TMAO) accumulate in PD patients. The objective was to explore the gut microbiota and their influence on uremic toxins in PD patients and healthy controls (HC). Fecal samples were collected from PD patients (n = 105) and HC (n = 102). 16S rRNA gene regions were sequenced for gut microbiota analysis. IS, PCS, and TMAO levels were measured using HPLC-MS. PD patients exhibited lower alpha diversity and altered gut microbiota composition compared to HC. At the genus level, PD patients showed increased abundance of opportunistic pathogenic bacteria, and decreased abundance of beneficial bacteria. Three Operational Taxonomic Units discriminated PD patients from HC. Phenylalanine metabolism increased in PD, whereas tryptophan metabolism was unaltered. Low serum PCS did not necessarily mean healthier due to the loss of alpha diversity, increased Proteobacteria and opportunistic pathogenic bacteria. High serum PCS was mainly caused by elevated p-cresol-producing bacteria, enriched amino acid related enzymes, and enhanced sulfur metabolism, rather than declined residual renal function. In patients with different urine volumes, the gut microbiota alpha diversity and composition were unaltered, but serum IS and TMAO were significantly elevated in anuric patients. In conclusion, the gut microbiota abundance, composition, and function were altered in PD patients, which increased the PCS levels. We provided a better understanding of the microbiota-metabolite-kidney axis in PD patients. Targeting certain bacteria could decrease the PCS levels, whereas preserving the residual renal function could reduce the IS and TMAO levels.

## Introduction

The gut microbiota is the largest microecosystem in our body (De Sordi et al., 2017). Gut microbiota dysbiosis is related to many diseases including acute kidney injury and chronic kidney disease (CKD) (Emal et al., 2017; Ren et al., 2020). Peritoneal dialysis (PD) is an effective renal replacement therapy administered to approximately 11% of end-stage renal disease patients undergoing dialysis. The long-term frequent inflow and outflow of glucose dialysate in the peritoneal cavity and uremia may change the abundance and composition of the gut microbiota. A few studies found that PD patients have altered gut microbial composition (Wang et al., 2012; Stadlbauer et al., 2017). However, the differences in the gut microbiota between PD patients and healthy controls (HC) are not well known.

Indoxyl sulfate (IS), P-cresyl sulfate (PCS), and trimethylamine-N-oxide (TMAO) are gut microbiota-derived uremic toxins, which can contribute to the progression and cardiovascular complications of CKD and PD (Zeisel and Warrier, 2017; Cosola et al., 2018). Since IS and PCS are protein-bound uremic toxins, they are hardly removed through PD (Vanholder et al., 1999). Uremic toxins accumulate in PD patients due to reduced renal function and limited removal of uremic toxins through dialysis (Viaene et al., 2014). We hypothesized that the gut microbiota in PD could accelerate the generation of uremic toxins.

In this study, we aimed to compare the gut microbiota of PD patients with that of HC by conducting16S rRNA gene sequencing of fecal samples. We also investigated the correlations between gut microbiota, serum uremic toxins, and urine volumes in PD patients.

## Methods

### Participants

In this case-control study, we enrolled 105 continuous ambulatory PD patients using glucose dialysate (Dianeal, Baxter) with dialysis vintage > 6 months, treated in Zhongshan Hospital from February 2018 to July 2019, and 102 healthy controls (HC) who got their annual physical examination done in our hospital. All participants were Han nationality and older than 18 years old. Exclusion criteria included: (1) gastrointestinal diseases; (2) any specific drug use in the previous three months, including antibiotics, probiotics, prebiotics, synbiotics, proton pump inhibitors, and immunosuppressive agents; (3) severe liver diseases, hepatitis; (4) tumor; (5) other immunological or autoimmune disorders. The study was approved by the Ethical Committee of Zhongshan Hospital, Fudan University (Approval No.: B2017-108R). All participants provided written informed consent.

### Fecal and serum sample collection

Stool samples were collected from the participants by spontaneous evacuation, transported to our laboratory on dry ice, divided into four equal parts of 200 mg, and immediately stored at −80°C for further analysis. Blood was collected in the morning pre-prandial. After centrifugation (3000×*g*, 10 min), the serum was immediately frozen at −80°C until use.

### Clinical assessment

Serum creatinine, blood urea nitrogen, uric acid, albumin, hemoglobin, triglyceride, total cholesterol, calcium, phosphorus, and C-reactive protein (CRP) levels were measured using standard methods followed in Zhongshan Hospital. Body mass index (BMI) was calculated using the formula: BMI = kg/m^2^. The CKD-EPI equation was used to calculate the estimated glomerular filtration rate (eGFR) (Levey et al., 2009). IS, PCS, and TMAO levels were measured using high performance liquid chromatography mass spectrometry (HPLC-MS) as previously described (Cao et al., 2015). The normalized protein catabolic rate (nPCR) was calculated using the PD adequest 2.0 software (Baxter Healthcare, Norfolk, UK) as a measure of daily protein intake. Kt/V (kidney) was used to evaluate the renal excretory function, and Kt/V (peritoneum) was used to monitor PD treatment efficiency based on urea clearance (Wang and Wang, 2015). A questionnaire was administered to PD patients (n = 40) and HC (n = 20) to collect information about the number of bowel movements per week and the Bristol Stool Scale assessing constipation (harder stool).

### 16S rRNA microbial profiling analysis and functional annotation

Bacterial DNA was extracted from stool samples using the E.Z.N.A.^®^ Stool DNA Kit following the manufacturer’s instructions (Omega Bio-tek, Inc., GA). The V3-V4 variable regions of the microbial 16S rRNA gene were amplified using the following primers: 341F (CCTACGGGNGGCWGCAG) and 805R (GACTACHVGGGTATCTAATCC). The products from different samples were indexed, mixed at equal ratios, and sequenced using an Illumina Miseq platform (Illumina Inc., USA) by Shanghai Mobio Biomedical Technology Co. Ltd, China (Caporaso et al., 2012).

After raw data extraction using USEARCH 8.0, an average of 35727 ± 8763 reads per sample was obtained. The sequences were classified into Operational Taxonomic Units (OTUs) clusters based on 97% similarity (3% divergence). Each 16S rRNA gene sequence was annotated using the RDP Classifier (http://rdp.cme.msu.edu/), referring to the Silva (SSU123) 16S rRNA database.

Alpha diversities are presented as the Shannon, Chao, and Ace diversity indices using the “vegan” package in R. Principal Coordinate Analysis (PCoA) plots and non-metric multidimensional scaling (NMDS) analysis were generated to visualize the unweighted UniFrac distances using the QIIME pipeline in R. The differential taxa between the PD and HC groups were detected using the linear discriminant analysis (LDA) effect size (LEfSe) (LDA score = 2 as the cut-off value).

Kyoto Encyclopedia of Genes and Genomes (KEGG) (Kanehisa et al., 2017) analysis was used to predict functional pathway information as categorized using the phylogenetic investigation of communities by reconstruction of unobserved states (PICRUSt) algorithm (Langille et al., 2013).

### Statistical analysis

All continuous data are presented as mean ± SD. Categorical variables are expressed as percentages. The student’s *t*-test or one-way ANOVA with the Bonferroni multiple comparison test was adopted to compare continuous variables. The Chi-squared test was used to compare categorical variables. Univariate correlation was assessed using Spearman’s correlation analyses. A P-value of <0.05 (two tailed) was considered statistically significant. SPSS (version 23.0) and GraphPad Prism (version 7.0) were used for statistical analysis.

## Results

### Differences in the serum metabolomes of PD patients and HC

The participants in the PD and HC groups were matched for age, gender, and BMI. Compared with the HC, the renal function in the PD group was significantly decreased as shown by the serum creatinine and eGFR. IS, PCS, and TMAO levels were remarkably increased in PD patients. The inflammatory biomarker CRP was also enhanced in PD (**Table 1**).

**Table 1:** Clinical characteristics of peritoneal dialysis patients and healthy controls.

Characteristic	PD (n=105)	HC (n=102)	P-value
Age, years	56.79 ± 13.74	54.20 ± 11.25	0.139
Gender, male/female, n	52/53	51/51	1.000
Body mass index, kg/m^2^	23.02 ± 4.16	22.90 ± 2.13	0.801
Serum creatinine, μmol/L	857.46 ± 257.27	74.29 ± 14.39	<0.001
Blood urea nitrogen, mmol/L	18.38 ± 4.92	4.51 ± 0.92	<0.001
Uric acid, umol/L	371.01 ± 96.69	344.07 ± 70.55	0.023
eGFR, ml/min/1.73m^2^	5.13 ± 1.66	90.70 ± 13.67	<0.001
Albumin, g/L	35.00 ± 4.89	43.53 ± 2.71	<0.001
RBC, ×10^12^/L	3.52 ± 0.76	4.61 ± 0.49	<0.001
Hemoglobin, g/L	105.67 ± 19.80	139.86 ± 16.86	<0.001
WBC, ×10^9^/L	7.22 ± 2.19	5.55 ± 1.11	<0.001
Platelet, ×10^9^/L	205.53 ± 80.08	201.66 ± 46.1	0.672
Triglyceride, mmol/L	1.94 ± 1.16	1.66 ± 0.95	0.061
Total cholesterol, mmol/L	4.38 ± 1.13	4.78 ± 0.90	0.005
Calcium, mmol/L	2.48 ± 1.98	2.28 ± 0.10	0.301
Phosphorus, mmol/L	1.57 ± 0.45	1.17 ± 0.15	<0.001
PCS, μg/mL	20.68 ± 15.54	0.63 ± 0.81	<0.001
IS, μg/mL	27.00 ± 14.81	0.35 ± 0.26	<0.001
TMAO, μg/mL	5.14 ± 3.45	0.19 ± 0.28	<0.001
CRP, mg/L	7.53 ± 12.88	1.00 ± 1.56	<0.001
Bristol Stool Scale score	4.1 ± 1.32	4.2 ± 0.77	0.755
Number of weekly bowel movements	11.50 ± 5.22	7.05 ± 2.01	<0.001
Hypertension, n (%)	100 (95.24)	NO	–
Diabetes, n (%)	23 (21.90)	NO	–

eGFR, estimated glomerular filtration rate; RBC, red blood cells; WBC, white blood cells; PCS, p−cresyl sulfate; IS, Indoxyl sulfate; TMAO, trimethylamine-N-oxide; CRP, C-reactive protein. Values were presented as mean ± SD or n (%).

### Decreased gut microbiota alpha-diversity in PD patients

**Figure 1:**
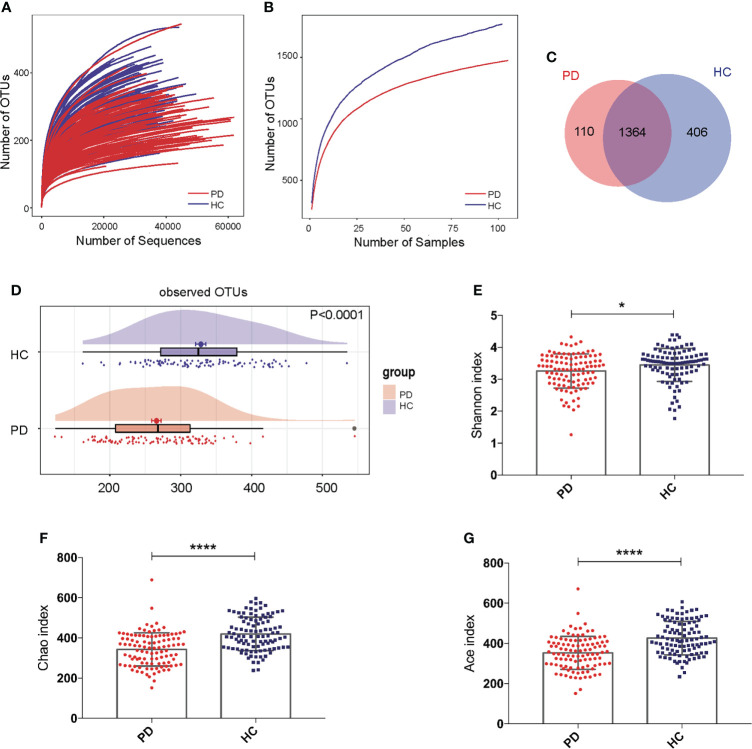
Gut microbiota alpha-diversity was decreased in peritoneal dialysis (PD) patients. **(A, B)** Rarefaction analysis between the number of OTUs and the number of sequences or samples in PD patients and healthy controls (HC) showing that the data were qualified. **(C)** Venn diagram showing the overlapping OTUs and the unique OTUs in PD patients and HC. **(D)** The OTUs were significantly reduced in PD patients compared to HC. **(E–G)** The Shannon, Chao and Ace indices were remarkably lower in PD patients than in HC. PD, peritoneal dialysis; HC, healthy controls; OTUs, operational taxonomic units. *P <0.05; ****P <0.0001.

The rarefaction curve showed that with the increasing number of sequences or samples, the number of OTUs reached a plateau, meaning the data were qualified and the sample size was sufficient (**Figures 1A, B**). The Venn diagram showed that 110 OTUs were unique for PD, 406 OTUs were unique for HC, and 1364 OTUs were shared by both groups (**Figure 1C**). The OTUs were significantly reduced in PD patients compared to those of HC (P<0.0001; **Figure 1D**). The Shannon (P <0.05), Chao (P <0.0001), and Ace indices (P <0.0001) were significantly lower in PD patients compared to that in HC (**Figures 1E–G**).

### Altered gut microbiota composition in PD patients

**Figure 2:**
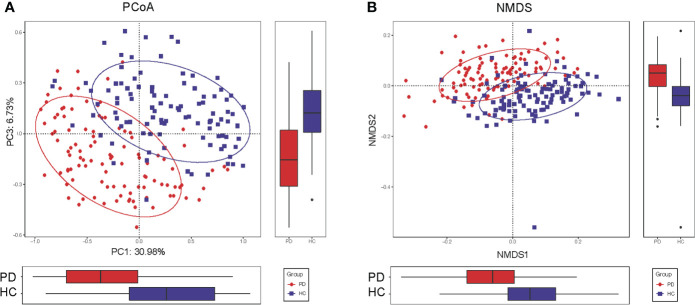
Altered gut microbiota composition in PD patients. **(A)** The PCoA of microbiota composition in PD patients and HC. **(B)** The NMDS analysis of microbiota composition in PD patients and HC. The circles cover samples near the gravity center in each group. PD, peritoneal dialysis; HC, healthy controls; PCoA, principal coordinate analysis. NMDS, non-metric multidimensional scaling.

To compare the beta diversity, the PCoA and NMDS analysis based on unweighted UniFrac distance analysis, displayed the microbiome space between the samples. A significant difference was observed between the PD and HC groups with the distribution of the OTUs from PC1 (30.98%), PC2 (20.20%), and PC3 (6.73%) (P<0.001, **Figure 2A**). The NMDS analysis also revealed a separation trend of PD and HC from NMDS1 and NMDS2 (P<0.001, **Figure 2B** **).**

### Differences in gut microbiota abundance between PD and HC

**Figure 3:**
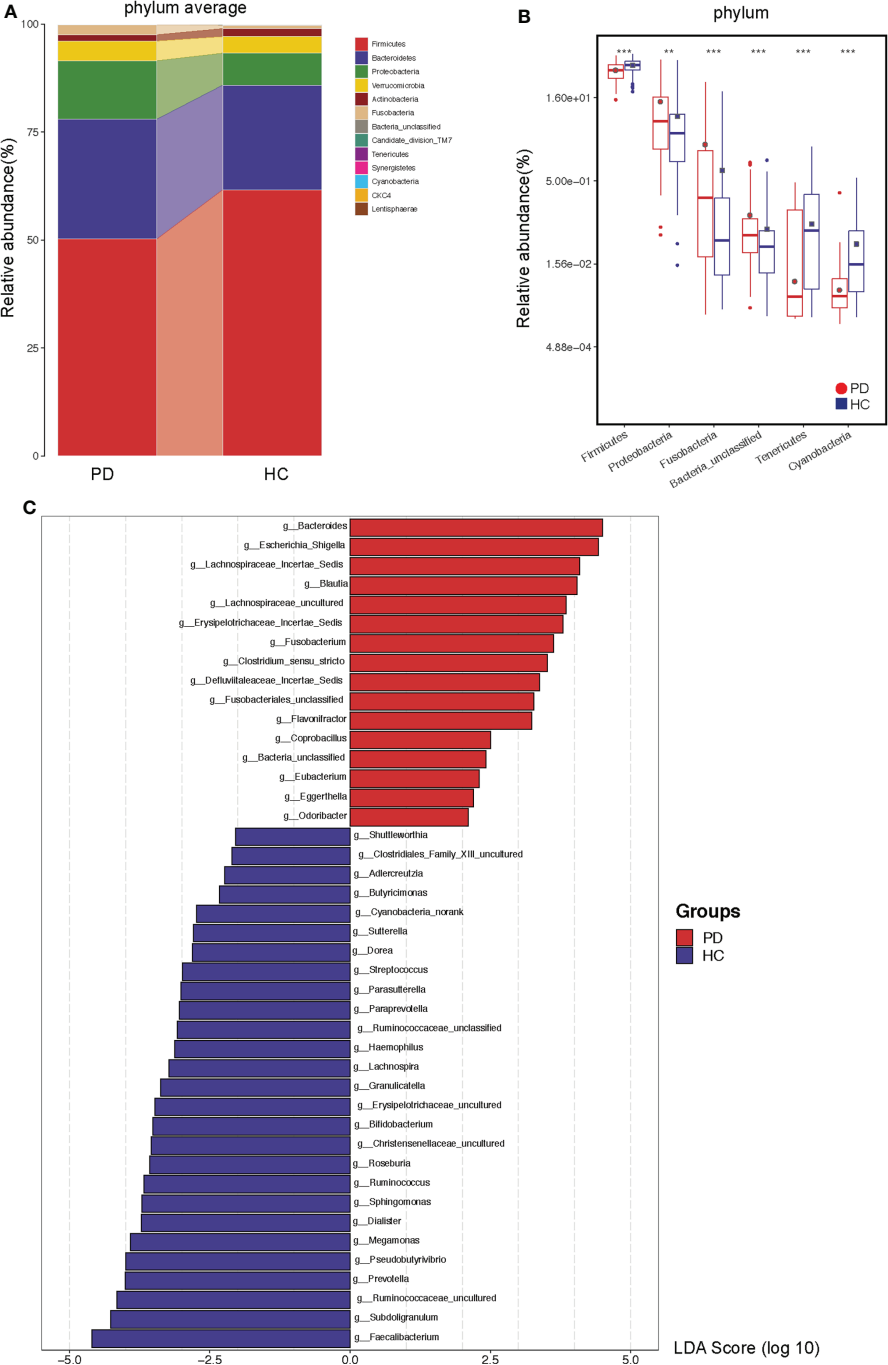
The different abundance of gut microbiota in peritoneal dialysis (PD) patients and healthy controls (HC) at the phylum and genus levels. **(A)** The average microbial compositions in PD patients and HC at the phylum level. **(B)** The significantly different phyla between PD patients and HC. **(C)** The significantly different genera between PD patients and HC at the genus level. **P <0.01, ***P <0.001. PD, peritoneal dialysis; HC, healthy controls.

The top 50 differentially abundant OTUs between PD patients and HC were presented in a heatmap (**Supplementary Figure S1**). Cladograms generated by LEfSe indicated the differences in the predominant bacteria between the PD and HC groups (**Supplementary Figure S2**). Furthermore, most gut microbiota fall into three phyla: Firmicutes, Bacteroidetes and Proteobacteria in PD (~50%, 28%, 14%, respectively) and HC (~62%, 24%, 7%, respectively) (**Figure 3A**). Proteobacteria (P <0.01), Fusobacteria (P <0.001), and Bacteria unclassified (P <0.001) were significantly increased, while Firmicutes (P <0.001), Tenericutes (P <0.001), and Cyanobacteria (P <0.001) were significantly decreased in PD patients compared to their levels in HC (**Figure 3B**). At the genus level, 16 and 27 genera were significantly increased and decreased, respectively, in PD compared to their levels in HC (LDA >2). We found a significant enrichment of opportunistic pathogens including *Bacteroides*, Escherichia Shigella, and *Flavonifractor*. Furthermore, a depletion in the abundance of beneficial bacteria, such as short-chain fatty acids (SCFA)-producing bacteria, including acetate-producing *Bifidobacterium*, butyrate-producing *Faecalibacterium*, *Subdoligranulum, Roseburia, Erysipelotrichaceae, Lachnospiraceae*, and *Clostridiales* (Lin and Hung, 2020) was observed in PD patients compared to that in HC (**Figure 3C**). Moreover, p-cresol-producing bacteria were enriched in PD, including *Fusobacterium, Clostridium*, and *Eubacterium*; *Bacteroides* and *Fusobacterium* are indole-producing bacteria (Li et al., 2020). They could lead to an increased production of PCS and IS. At the species levels, the similar results were observed (**Supplementary Figure S3**). Meanwhile, *Blautia* (r = 0.262, P = 0.008) and *Fusobacterium* (r = 0.233, P = 0.019) were associated with CRP. Thus, PD status influenced the relative quantity of certain gut microbiota and strengthened the importance of bacteria in systemic inflammation or accumulation of uremic toxins in PD patients.

### Predictive function analysis

**Figure 4:**
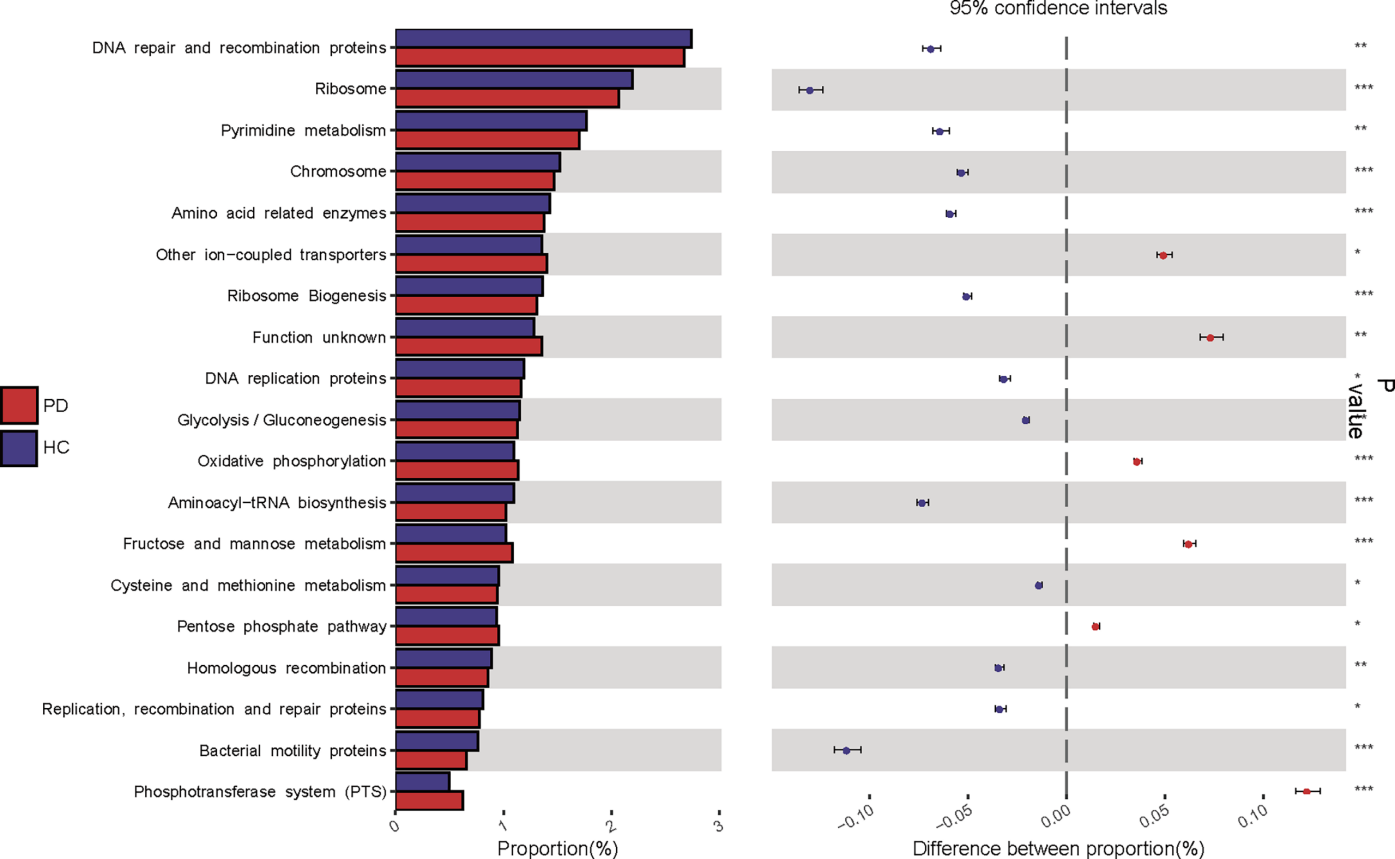
Kyoto Encyclopedia of Genes and Genomes (KEGG) pathway analysis predicted gut microbial functions in peritoneal dialysis (PD) patients and healthy controls (HC) at level three. PD, peritoneal dialysis; HC, healthy controls. *P <0.05; **P <0.01, ***P <0.001.

KEGG pathway analysis was conducted to predict bacterial functions in the PD and HC groups. At level three, we found that 22 predicted gut microbial functions, including oxidative phosphorylation, fructose and mannose metabolism, and pentose phosphate pathway, were significantly increased; while 34 functions, including DNA repair and recombination proteins, glycolysis and gluconeogenesis, were significantly decreased in PD patients compared with those of HC (P <0.05, LDA >2. **Figure 4**; **Supplementary Data S1**). The long-term effects of high glucose dialysate in the peritoneal cavity of PD patients may explain the disordered glucose metabolism. Notably, bacterial motility proteins, bacterial chemotaxis, flagellar assembly, and peptidoglycan biosynthesis were reduced in PD patients (**Supplementary Data S1**). Although IS was a tryptophan metabolite, no significant difference in tryptophan metabolism was observed between the two groups (P >0.05). Furthermore, the enriched phenylalanine metabolism (P = 0.043, LDA < 2) in PD confirmed that gut microbiota play a more important role in the metabolism of PCS than that of IS.

### Validity of gut microbiota in discriminating PD

**Figure 5:**
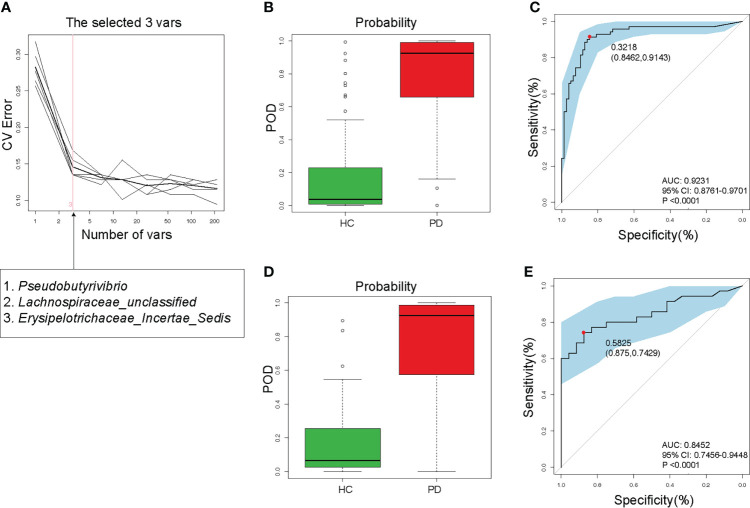
Validity of gut microbiota in discriminating peritoneal dialysis (PD) patients from healthy controls (HC). **(A)** Three OTUs (*Pseudobutyrivibrio*, *Lachnospiraceae*, and *Erysipelotrichaceae*) were selected as markers by random forest models. **(B)** The POD value was significantly increased in PD patients than in HC in the training cohort. **(C)** The AUC value was 0.9231 in the training cohort. **(D)** The POD value was remarkably increased in PD patients in the testing cohort. **(E)** The AUC value was 0.8452 in the testing cohort. PD, peritoneal dialysis; HC, healthy controls; CV, cross-validation; POD, probability of disease; AUC, area under curve; CI, confidence interval.

To detect unique OTUs markers of PD patients, we conducted fivefold cross-validation on a random forest model between 105 PD patients and 102 HC samples. Three OTUs (*Pseudobutyrivibrio*, *Lachnospiraceae*, and *Erysipelotrichaceae*) were selected as the optimal markers of PD with minor CV error (**Figure 5A**). In the training cohort, the probability of disease (POD) value calculated using the three optimal OTUs was significantly higher in PD patients (approached 1) compared to that of HC (approached 0, **Figure 5B**). The receiver operating characteristics (ROC) curve showed that the area under the curve (AUC) of the POD value was 0.9231 (**Figure 5C**). In the testing cohort, the POD value was also significantly increased in PD (**Figure 5D**), and the AUC was 0.8452 (**Figure 5E**). Although PD does not require specific gut microbiota as diagnostic markers, the high value of AUC suggested that PD could be distinguished from HC by the three OTUs with high specificity and sensitivity. Measuring the variation of the 3 OTUs described in **Figure 5A** may help to monitor the effectiveness of PD treatment (such as probiotics or synbiotics).

### Correlation between the gut microbiome and PCS in PD patients

**Figure 6:**
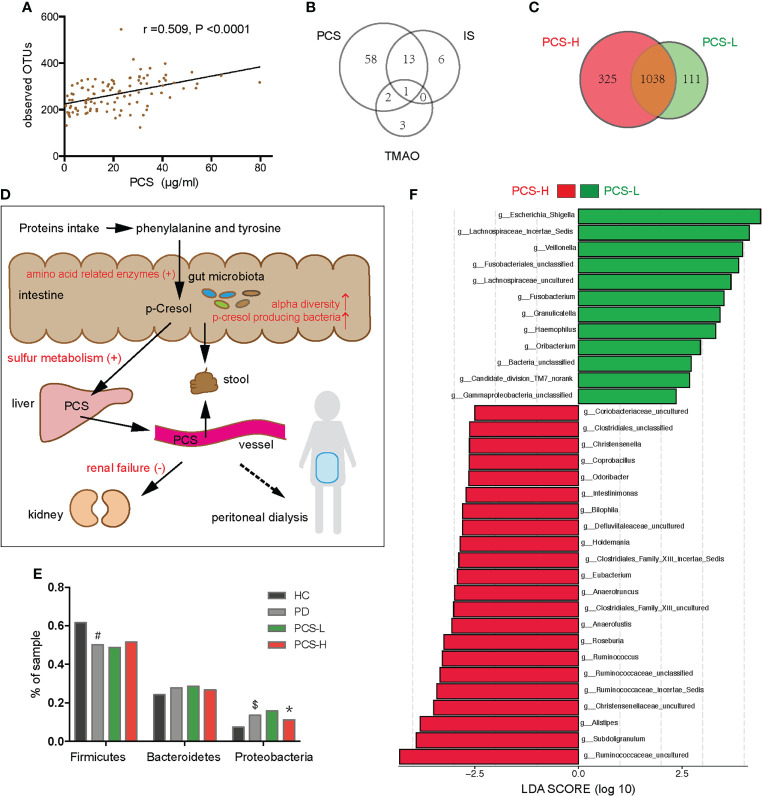
Correlation between the gut microbiota and p-cresyl sulfate (PCS) levels. **(A)** Correlation between PCS levels and the observed OTUs. **(B)** The number of OTUs associated with PCS, IS and TMAO levels. **(C)** Venn diagram showing the overlapping OTUs and the unique OTUs between the PCS-L and PCS-H subgroups. **(D)** The PCS metabolic pathway (black fonts). PCS is mainly derived from phenylalanine and tyrosine, metabolized by gut microbiota in the intestine, sulfated by the liver, and excreted by the kidney or stool (the solid line), but hardly removed by peritoneal dialysis (the dotted line). The red fonts indicate that many factors elevate the serum PCS level. **(E)** *Firmicutes*, *Bacteroidetes*, and *Proteobacteria* in four groups. **(F)** The significantly different bacteria in the PCS-L and PCS-H subgroups at the genus level. PCS, p-cresyl sulfate; OTUs, operational taxonomic units; IS, Indoxyl sulfate; TMAO, trimethylamine-N-oxide; PCS-L, PCS-Low; PCS-H, PCS-High; PD, peritoneal dialysis; HC, healthy controls. ^#^P <0.001 vs HC, ^$^P <0.01 vs HC, *P <0.05 vs PCS-L.

In PD patients, alpha-diversity, as measured by Shannon, Chao, Ace indices, and observed OTUs, was positively correlated with PCS in PD patients (**Table 2**; **Figure 6A**). There was a weak correlation between alpha-diversity and IS or phosphorus levels (r <0.3). The alpha-diversity did not correlate with TMAO levels and other variables. PCS was associated with 74 OTUs, IS with 20 OTUs, and TMAO with six OTUs in PD patients (**Figure 6B**). Among them, 14 OTUs were both associated with PCS and IS. Hence, the degree of microbiota disorder in PD patients was more closely related to PCS than that to IS and TMAO.

**Table 2:** Correlation between alpha diversity and variables.

	Shannon index		Chao index		Ace index			Observed OTUs	
	r	P-value	r	P-value	r	P-value		r	P-value
Age	0.107	0.278	0.135	0.170	0.082	0.407		0.152	0.121
Dialysis vintage	0.005	0.963	-0.045	0.651	0.001	0.989		-0.105	0.288
BMI	-0.153	0.120	0.047	0.637	0.022	0.826		0.046	0.639
PCS	0.306	0.002	0.512	<0.001	0.435	<0.001		0.509	<0.001
IS	0.106	0.280	0.259	0.008	0.206	0.035		0.212	0.030
TMAO	-0.044	0.658	0.110	0.263	0.028	0.776		0.027	0.783
eGFR	0.039	0.694	-0.029	0.770	-0.050	0.614		0.017	0.862
24h urine volumns	-0.035	0.723	0.101	0.303	0.048	0.629		0.135	0.170
Creatinine	-0.031	0.756	0.021	0.828	0.020	0.839		-0.028	0.776
Calcium	0.041	0.675	0.093	0.344	0.090	0.363		0.095	0.337
Phosphorus	-0.227	0.020	-0.062	0.528	-0.080	0.420		-0.076	0.439
Serum albumin	0.009	0.927	0.100	0.312	0.098	0.320		0.095	0.336
CRP	-0.020	0.843	0.050	0.620	0.064	0.527		0.029	0.771

OTUs, operational taxonomic units; BMI, body mass index; PCS, p-cresyl sulfate; IS, Indoxyl sulfate; TMAO, trimethylamine-N-oxide; eGFR, estimated glomerular filtration rate; CRP, C-reactive protein.

The metabolic pathway of PCS is shown in **Figure 6D**. To clarify the relationship between PCS and gut microbiota, we divided the PD patients into PCS-low (L) (PCS ≤ 18.7 μg/ml, n = 53) and PCS-high (H) (PCS > 18.7 μg/ml, n = 52) subgroups according to the PCS median concentration and compared the clinical data in these subgroups (**Table 3** **).** Here, two subgroups had similar protein intake as evaluated by nPCR and albumin. No difference was observed in the eGFR, urine volumes, Kt/V (kidney), and Kt/V (peritoneum), implying similar renal excretory function and PD treatment efficiency. The comorbidities and medicinal regimens also showed no significant difference between two subgroups (**Supplementary Table S1** **)**.

**Table 3:** Clinical data of PCS-L and PCS-H subgroups.

Characteristic	PCS-L (n=53)	PCS-H (n=52)	P-value
Age, years	55.51 ± 12.74	58.1 ± 14.69	0.337
Gender, male/female, n	28/25	24/28	0.560
Body mass index, kg/m^2^	23.64 ± 4.23	22.38 ± 4.03	0.117
Dialysis vintage, month	27.62 ± 24.32	29.63 ± 33.36	0.724
Kt/V (kidney)	0.55 ± 0.56	0.63 ± 0.66	0.522
Kt/V (pritoneum)	1.50 ± 0.47	1.55 ± 0.37	0.554
24h urine volumn, L	0.60 ± 0.53	0.73 ± 0.66	0.279
eGFR, ml/min/1.73m2	4.92 ± 1.63	5.34 ± 1.68	0.195
Serum creatinine, μmol/L	901.13 ± 273.60	812.94 ± 233.76	0.079
Blood urea nitrogen, mmol/L	18.37 ± 5.24	18.39 ± 4.61	0.981
Uric acid, μmol/L	374.34 ± 75.91	367.62 ± 114.74	0.723
Hemoglobin, g/L	104.83 ± 21.44	106.52 ± 18.14	0.664
Serum albumin, g/L	34.51 ± 4.66	35.50 ± 5.10	0.301
nPCR	0.89 ± 0.20	0.95 ± 0.28	0.208
Triglyceride, mmol/L	2.07 ± 1.27	1.80 ± 1.02	0.245
Total cholesterol, mmol/L	4.36 ± 1.21	4.39 ± 1.05	0.923
Calcium, mmol/L	2.25 ± 0.18	2.72 ± 2.80	0.226
Phosphorus, mmol/L	1.63 ± 0.41	1.51 ± 0.49	0.168
CRP, mg/L	6.21 ± 12.24	8.73 ± 13.42	0.327
Number of observed OTUs	239.19 ± 49.53	292.81 ± 73.65	<0.001
Shannon index	3.15 ± 0.46	3.37 ± 0.59	0.040
Chao index	310.65 ± 63.26	376.02 ± 87.74	<0.001
Ace index	324.62 ± 68.99	382.68 ± 84.66	<0.001
PCS, μg/mL	8.39 ± 5.36	33.2 ± 12.09	<0.001
IS, μg/mL	22.97 ± 13.23	31.11 ± 15.32	0.004
TMAO, μg/mL	5.02 ± 3.25	5.26 ± 3.66	0.723

eGFR, estimated glomerular filtration rate; PCS, p-cresyl sulfate; nPCR, normalized protein catabolic rate; CRP, C-reactive protein; PCS, p-cresyl sulfate; IS, Indoxyl sulfate; TMAO, trimethylamine-N-oxide. Values were presented as mean ± SD or n.

Notably, the alpha diversity of gut microbiota was significantly reduced in the PCS-L subgroup compared to PCS-H subgroup (**Table 3**). A Venn diagram showed that 111 OTUs were unique in the PCS-L subgroup, and 325 OTUs were unique in the PCS-H subgroup (**Figure 6C**), suggesting that patients with low serum PCS were not necessarily healthier due to a diminished bacterial abundance. Gut microbiota composition was also significantly altered in the two subgroups as demonstrated using PCoA and NMDS analysis (P<0.001, **Supplementary Figure S4**). The top three abundant phyla were Firmicutes, Bacteroidetes, and Proteobacteria in the PCS-L (~49%, 29%, 16%, respectively) and PCS-H subgroups (~52%, 27%, 11%, respectively, **Figure 6E**). Interestingly, the ratio of the three phyla in the PCS-H subgroup was much similar to that of HC. The significantly increased genera in PCS-H might contribute to increased PCS (**Figure 6F**). The p-cresol-producing bacteria *Ruminococcus*, *Roseburia*, and *Anaerococcus* enriched in PCS-H could accelerate PCS biosynthesis. Remarkably, the opportunistic pathogenic bacterium Escherichia Shigella was elevated in PCS-L. KEGG pathway analysis predicted bacterial functions in two subgroups at levels 1–3 (**Supplementary Figure S5**). As expected, amino acid-related enzymes (P = 0.003, LDA>2) and sulfur metabolism (P = 0.037, LDA>2) were increased in the PCS-H subgroup, which can explain the enhanced serum PCS and IS levels.

Furthermore, comparisons of the gut microbiota in terms of the IS and TMAO levels, showed that gut microbiome abundance and composition did not differ between the IS-low and IS-high subgroups divided by the median IS concentration (25.4 μg/ml). No difference was observed between the TMAO-low and TMAO-high subgroups divided by the median TMAO concentration (4.1 μg/ml), either. Thus, the disordered gut microbiota in PD was associated with serum PCS, rather than with IS or TMAO.

### Gut microbiome and urine volume correlations in PD patients

**Figure 7:**
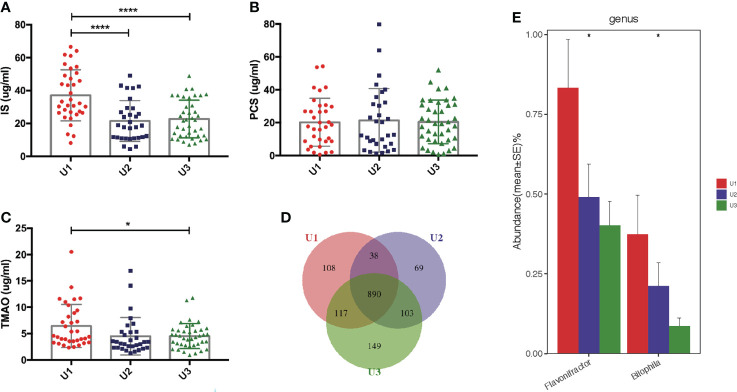
Correlation between the gut microbiota and urine volumes. **(A)** IS was remarkably increased in the U1 group compared with its levels in the U2 and U3 groups. **(B)** There was no significant difference of PCS levels among the three groups. **(C)** TMAO levels were higher in the U1 group than in the U3 group. **(D)** Venn diagram showing the overlapping OTUs and the unique OTUs among the U1, U2 and U3 groups. **(E)** *Flavonifractor* and *Bilophila* were gradually reduced in the U1, U2 and U3 groups. IS, Indoxyl sulfate; PCS, p-cresyl sulfate; TMAO, trimethylamine-N-oxide; OTUs, operational taxonomic units. *P <0.05; ****P <0.0001.

The mean of endogenous creatinine clearance rate and urea clearance rate, or urine volume was used to estimate residual renal function (RRF) (Lu et al., 2018). RRF is usually assessed by urine volumes in uremic patients. PCS levels showed no correlation with urine volumes (r = 0.054, P = 0.585). We assigned 105 PD patients into three subgroups according to urine volumes and investigated the levels of uremic toxins. Anuria was defined as 24-h urine volumes <100 mL. PD patients with 24-h urine volumes of <100 mL (n = 34), 100–1000 mL (n = 31), and >1000 mL (n = 40) were assigned to the U1, U2, and U3 groups, respectively. IS was found to be remarkably increased in the U1 group compared to U2 and U3 groups (both P <0.0001, **Figure 7A**). TMAO levels were higher in the U1 group compared to U3 group (P <0.05, **Figure 7C**). However, the PCS levels did not significantly differ among three groups (P >0.05, **Figure 7B**).

Significant differences in the alpha and beta diversities were not detected among the U1, U2, and U3 groups (**Supplementary Data S2**; **Supplementary Figure S6**). A Venn diagram showed that 108, 69 and 149 OTUs were unique in the U1, U2 and U3 groups, respectively (**Figure 7D**). At the genus level, the levels of *Flavonifractor* (r = −0.321, P <0.05) and *Bilophila* (r = −0.231, P <0.05) gradually decreased with the increasing urine volumes in the U1, U2, and U3 groups (**Figure 7E**). Whether *Flavonifractor* and *Bilophila* can produce IS or TMAO has never been reported. Hence, there was no correlation between urine volumes and microbiota diversity.

## Discussion

Many studies have concentrated on the disordered gut microbiota in CKD stages 1–5 or in hemodialysis patients, however, the relationship between gut microbiota and uremic toxins in PD patients has been rarely reported.

In this study, first, we compared the gut microbiota between PD patients and HC (summarized in **Supplementary Figure S7**). We found that the alpha diversity was significantly decreased in PD patients compared to HC, consistent with previous studies (Crespo-Salgado et al., 2016; Stadlbauer et al., 2017). Many factors such as dietary restrictions, drugs, dialysis treatment, and malnutrition may inhibit the growth of certain bacteria. The genera enriched in PD patients were mainly opportunistic pathogens, and p-cresol and indole-producing bacteria, proving a correlation between uremic toxins and gut microbiota in PD. SCFAs including acetate, propionate, and butyrate have protective effects in inflammatory responses, stabilizing the gut barrier, and attenuating bacterial translocation (De Andrés et al., 2018; Routy et al., 2018). Supplying SCFAs could reduce local and systemic inflammation and improve renal function (Andrade-Oliveira et al., 2015; Du et al., 2020). The decreased acetate producer *Bifidobacterium* and butyrate producers *Faecalibacterium* and *Subdoligranulum*, suggest a diminished ability to generate SCFAs and a potential gut barrier dysfunction and systemic inflammation in PD patients. Furthermore, three OTUs could accurately differentiate PD patients from HC.

Second, we compared the gut microbiota in different PD subgroups. Increased PCS levels were directly associated with cardiovascular events and mortality, and the inflammatory marker interleukin-6 and oxidative stress marker glutathione peroxidase (Meijers et al., 2010; Rossi et al., 2014). The gut microbiota directly increased serum PCS levels with increased alpha diversity, altered microbiota composition, higher abundance of p-cresol-producing bacteria, enriched amino acid related enzymes, and elevated sulfur metabolism. A previous study showed that PCS and TMAO levels were positively correlated with the Shannon index in HC (Wilmanski et al., 2019). We proved that the higher alpha diversity was only associated with the serum microbial metabolite PCS in PD patients. Targeting p-cresol-producing bacteria may diminish PCS in PD patients resulting in therapeutic benefits. Combined supplementation with prebiotics and probiotics could reduce serum PCS in CKD patients (Rossi et al., 2016). However, a randomized crossover study found that inulin-type fructan intervention could not decline PCS in PD patients (Li et al., 2020). Thus, it is imperative to reduce PCS levels by targeting certain gut microbiota in PD patients.

We found that low serum PCS was partly due to reduced alpha diversity and abundance of p-cresol-producing bacteria. Microbial diversity could be influenced by inflammatory and nutritional status to some extent (Lin and Hung, 2020), and primarily by intestinal physiology (Reese and Dunn, 2018). Although reduced alpha diversity leads to lower PCS levels, it could be related to diseases such as inflammatory bowel disease (Pascal et al., 2017; Wilmanski et al., 2019). The phylum Proteobacteria was significantly enriched in patients with low PCS levels. Increased Proteobacteria is a potential diagnostic signature of microbial dysbiosis and risk of inflammation and disease (Shin et al., 2015; Rizzatti et al., 2017). The elevated opportunistic pathogenic bacteria also associated with the occurrence of peritonitis (Doğukan et al., 2000; Feng et al., 2014). Those suggested that patients with lower PCS levels were not always healthier. Their gut microbiota should be analyzed. If their low PCS were due to a lack of α-diversity or disordered bacteria, they may be worse than patients with high PCS.

Finally, we investigated whether decreased renal function could increase serum PCS levels in anuric PD patients. Some researchers found that serum PCS increased with the decreased eGFR or residual renal function in CKD and PD patients (Meijers et al., 2010; Viaene et al., 2014). In contrast, others found that increasing serum PCS did not parallel with declining residual renal function in PD patients (Bammens et al., 2005; Pham et al., 2008), while IS and TMAO were reported to have a strong correlation with eGFR (Pham et al., 2008; Pelletier et al., 2019). Consistently, we found that PCS was not correlated with urine volumes in PD patients, but IS and TMAO were still related to residual renal function, suggesting the validity of our enrolled patients. The kidney clearance rate of IS was three times that of PCS (Poesen et al., 2013). Serum PCS levels did not change with urine volumes possibly due to its lower kidney clearance rate compared to IS, as well as the different diets and life styles among three groups. The gut microbiota did not significantly change among the U1, U2, and U3 groups. We propose that kidney excretion has a greater influence on serum IS and TMAO levels than their generation by gut microbiota. A recent study showed that impaired kidney function was the main contributor to the increased serum IS levels at different CKD stages (Gryp et al., 2020). Our findings proved that increased IS and TMAO levels were mainly due to the loss of residual renal function in PD patients. The need to focus more on the production mechanisms of PCS rather than on its renal elimination has been emphasized through this study.

Our study had several limitations. Compared with 16S rRNA analysis, shotgun metagenome sequencing can provide a more accurate description of the gut microbiota composition and function. Furthermore, we did not include fiber intake and lifestyle assessments or measured the PCS level in urine, stool, and dialysate, which may have limited the interpretations of our results.

## Conclusion

The gut microbiota abundance, composition, and function were altered in PD patients. Our study provided novel insights into the relationship between gut microbiota and uremic toxins in PD patients. The gut microbiota might provide therapeutic possibilities for preventing the generation of PCS. Moreover, preserving residual renal function could reduce the IS and TMAO levels.

## Data availability statement

The raw Illumina read data for all samples were deposited in the European Bioinformatic Institute database under the accession code PRJNA682853 and PRJNA687563.

## Ethics statement

The studies involving human participants were reviewed and approved by Zhongshan Hospital, Fudan University. The patients/participants provided their written informed consent to participate in this study.

## Author contributions

MB designed the experiments and wrote the manuscript. MB and PZ collected samples and analyzed data. SG was involved in analyzing the data. JZ and JJ helped interpret data. XD and XY designed and supervised all experiments, and edited the manuscript. All authors contributed to the article and approved the submitted version.

## Funding

This work was funded by the National Natural Science Foundation of China (81970667) and the Development Plan of Top Young Talents in Shanghai (2018).

## Conflict of interest

The authors declare that the research was conducted in the absence of any commercial or financial relationships that could be construed as a potential conflict of interest.

## Publisher’s note

All claims expressed in this article are solely those of the authors and do not necessarily represent those of their affiliated organizations, or those of the publisher, the editors and the reviewers. Any product that may be evaluated in this article, or claim that may be made by its manufacturer, is not guaranteed or endorsed by the publisher.
