# Supplementary material for: Altered gut microbiota and gut-derived p-cresyl sulfate serum levels in peritoneal dialysis patients
Source: Front Cell Infect Microbiol. 2022 Sep 27;12:639624. doi: 10.3389/fcimb.2022.639624 (PMC9551184; doi:10.3389/fcimb.2022.639624)
Supplement: Supplementary file 1 [file DataSheet_1.zip › All supplementary Figure and Data/Supplementary Table 1. The comorbidities and medications between PCS-L and PCS-H.docx]

**Supplementary Table 2.** The comorbidities and medications between PCS-L and PCS-H

|  | PCS-L (n=53) | PCS-H (n=52) | P-value |
| --- | --- | --- | --- |
| **Comorbidities, n (%)** |  |  |  |
| Hypertension | 50 (94.3) | 50 (96.2) | 1.000 |
| Diabetes | 13 (24.5) | 10 (19.2) | 0.638 |
| **Medications, n (%)** |  |  |  |
| ACEI/ARB | 30 (56.6) | 21 (40.4) | 0.120 |
| Calcium channel blockers | 42 (79.2) | 42 (80.8) | 1.000 |
| Beta-blocker | 25 (47.2) | 30 (57.7) | 0.331 |
| Alpha-blocker | 3 (5.7) | 8 (15.4) | 0.123 |
| Statin | 10 (18.9) | 13 (25.0) | 0.487 |
| EPO | 28 (52.8) | 37 (71.2) | 0.071 |
| Ferrous succinate | 32 (60.4) | 36 (69.2) | 0.415 |

ACEI/ARB, angiotensin converting-enzyme inhibitor / angiotensin receptor blocker; EPO, erythropoietin.
