# Supplementary figures and images for: Altered gut microbiota and gut-derived p-cresyl sulfate serum levels in peritoneal dialysis patients
Source: Front Cell Infect Microbiol. 2022 Sep 27;12:639624. doi: 10.3389/fcimb.2022.639624 (PMC9551184; doi:10.3389/fcimb.2022.639624)

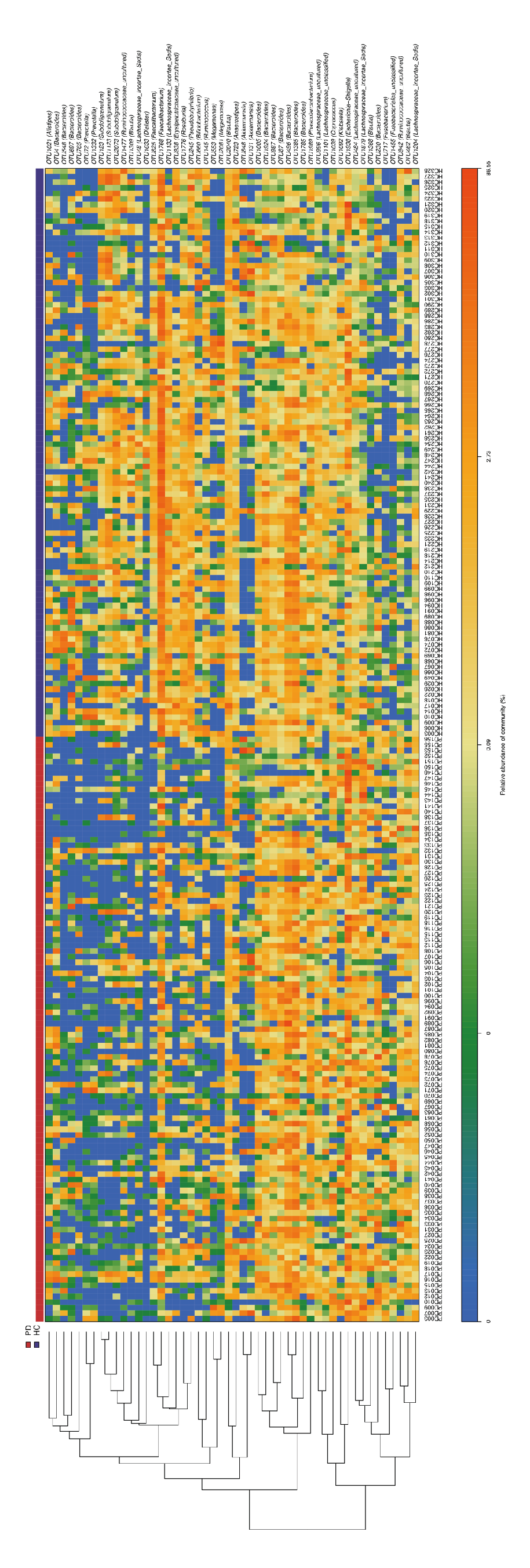

Supplement: Supplementary file 1 [file DataSheet_1.zip › All supplementary Figure and Data/s1 169*235.tif]

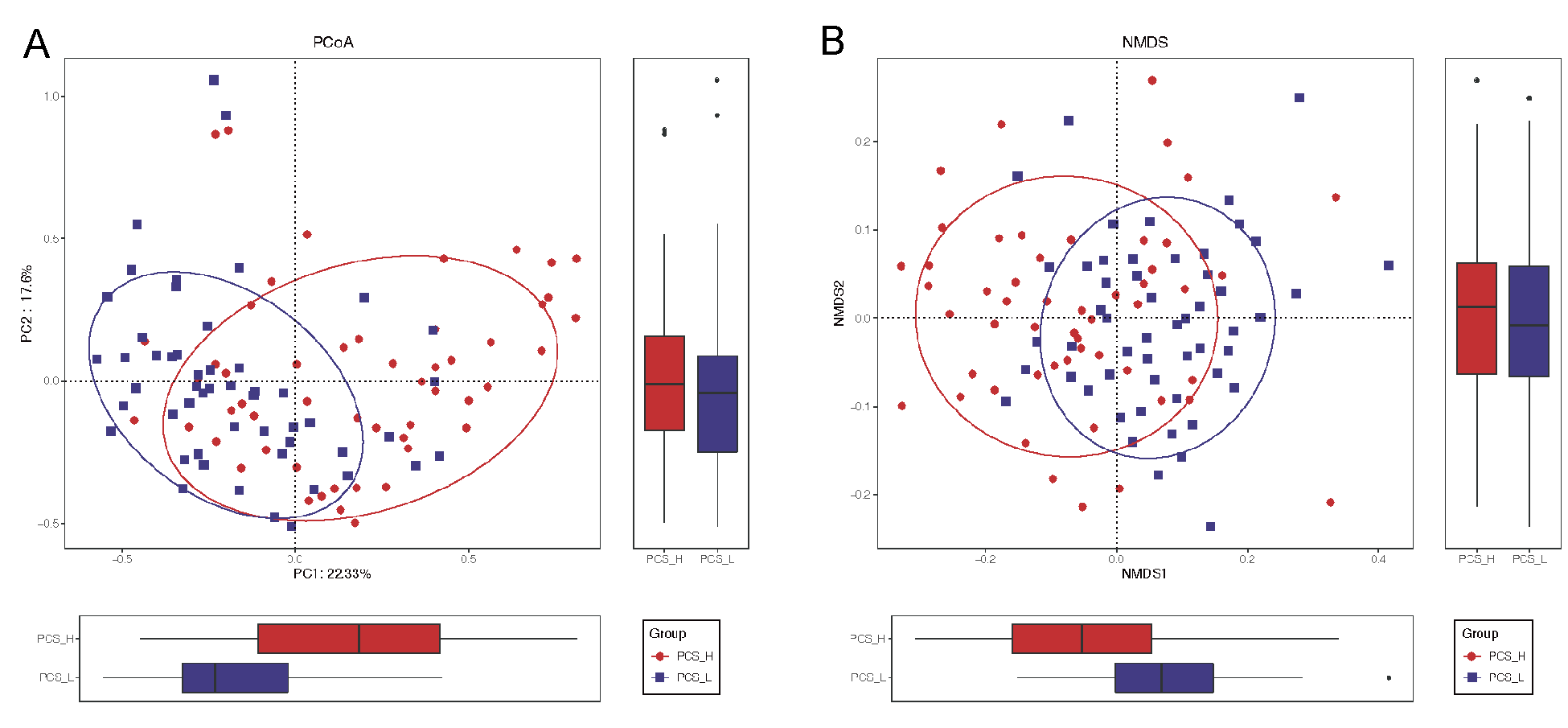

Supplement: Supplementary file 1 [file DataSheet_1.zip › All supplementary Figure and Data/s4 169*235.tif]

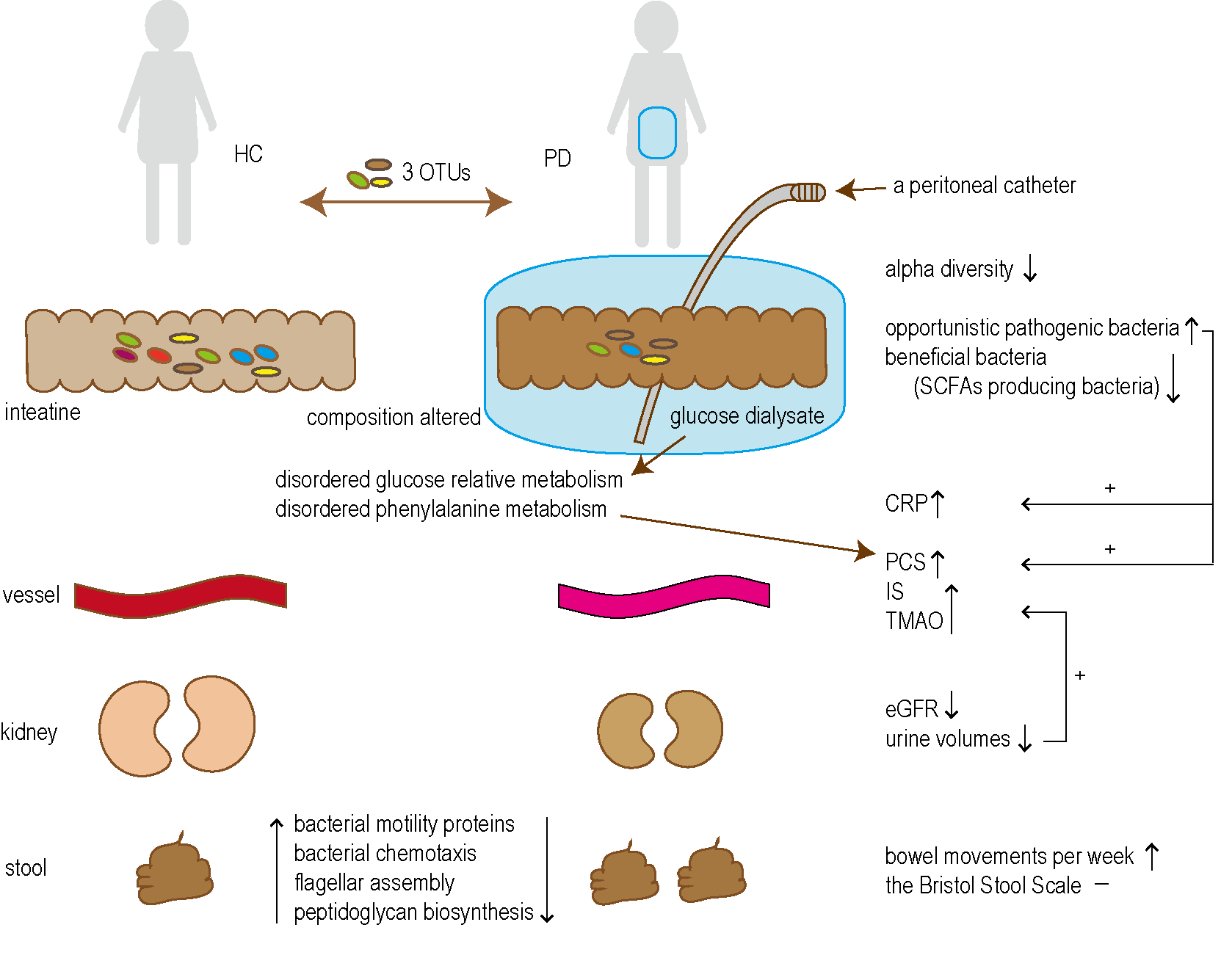

Supplement: Supplementary file 1 [file DataSheet_1.zip › All supplementary Figure and Data/s7 169*235.tif]

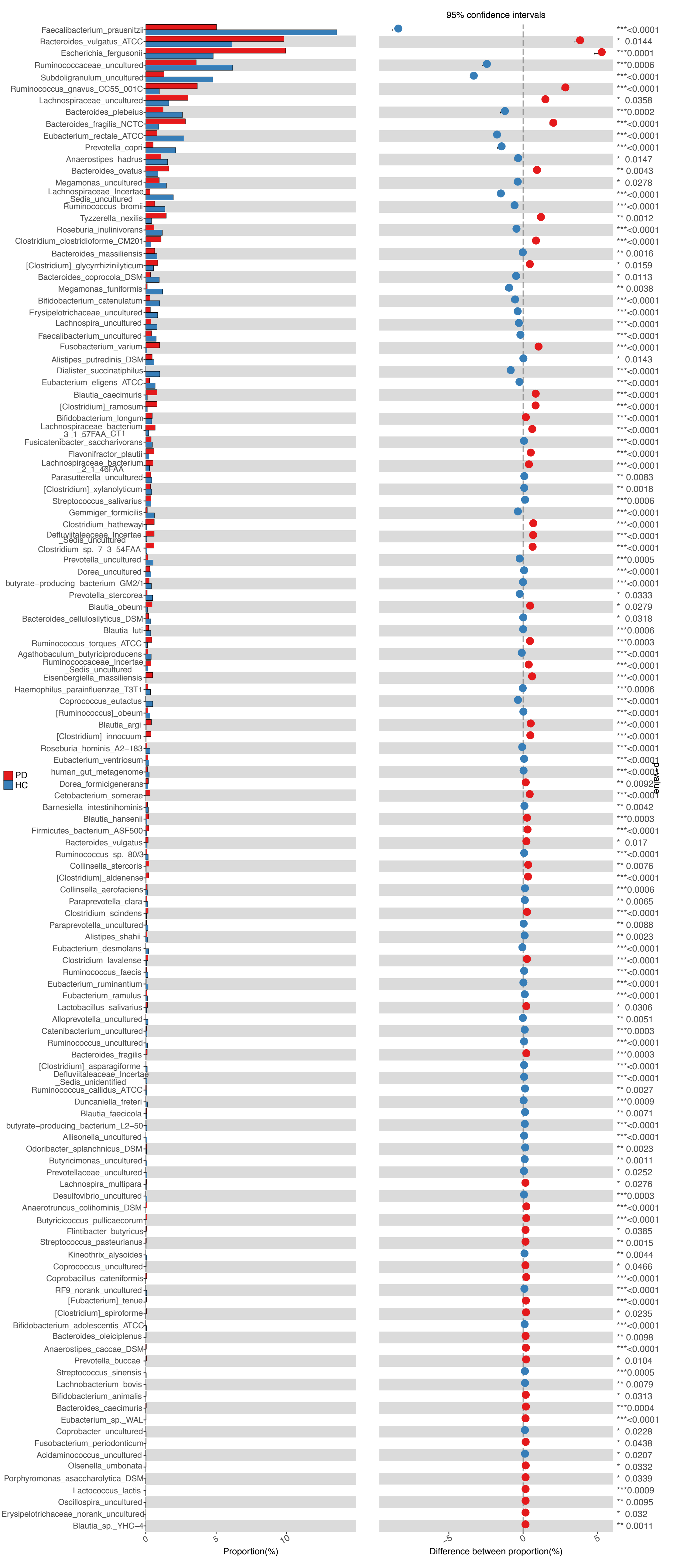

Supplement: Supplementary file 1 [file DataSheet_1.zip › All supplementary Figure and Data/s3.species.pdf]

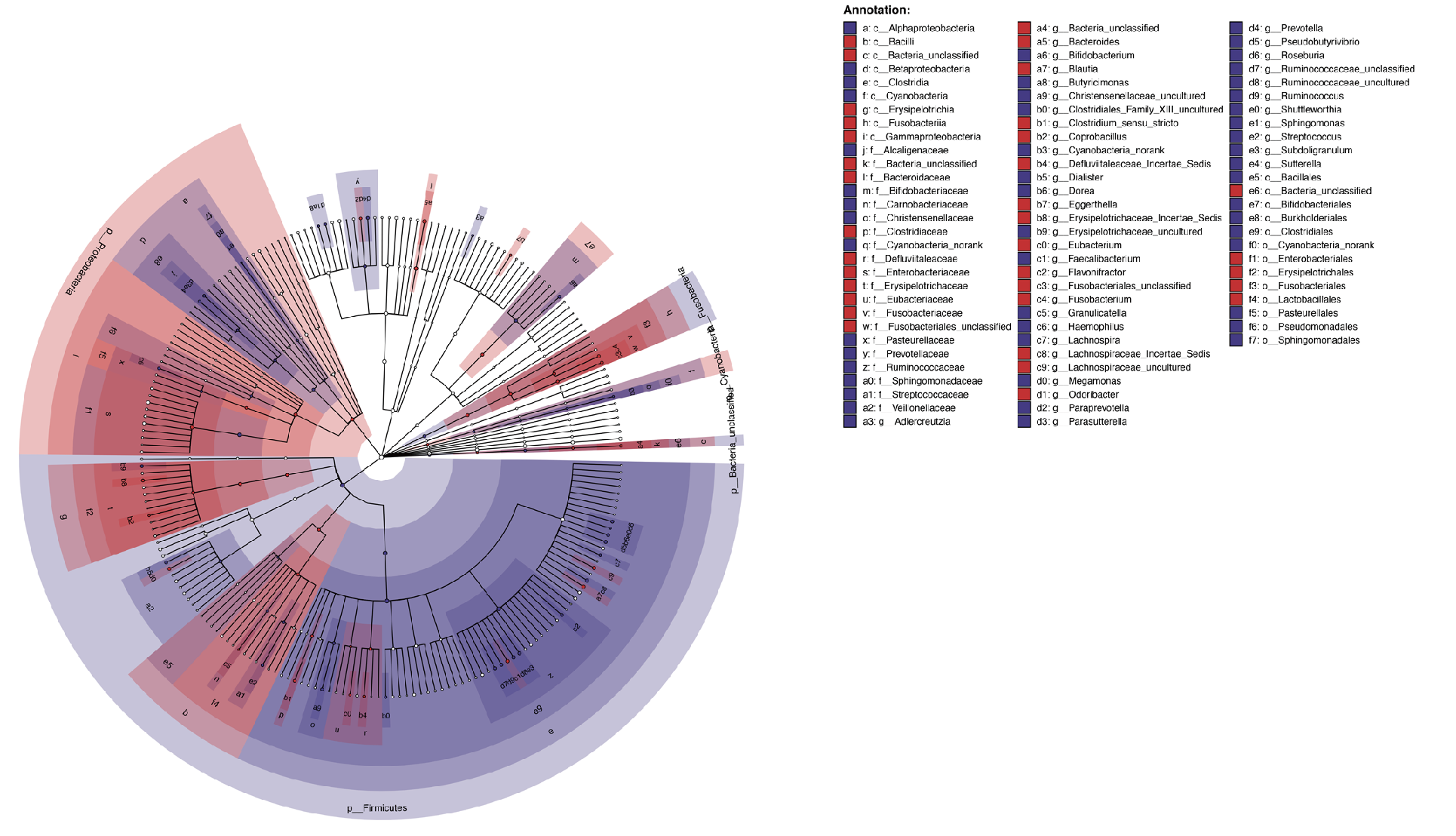

Supplement: Supplementary file 1 [file DataSheet_1.zip › All supplementary Figure and Data/s2 169*235.tif]

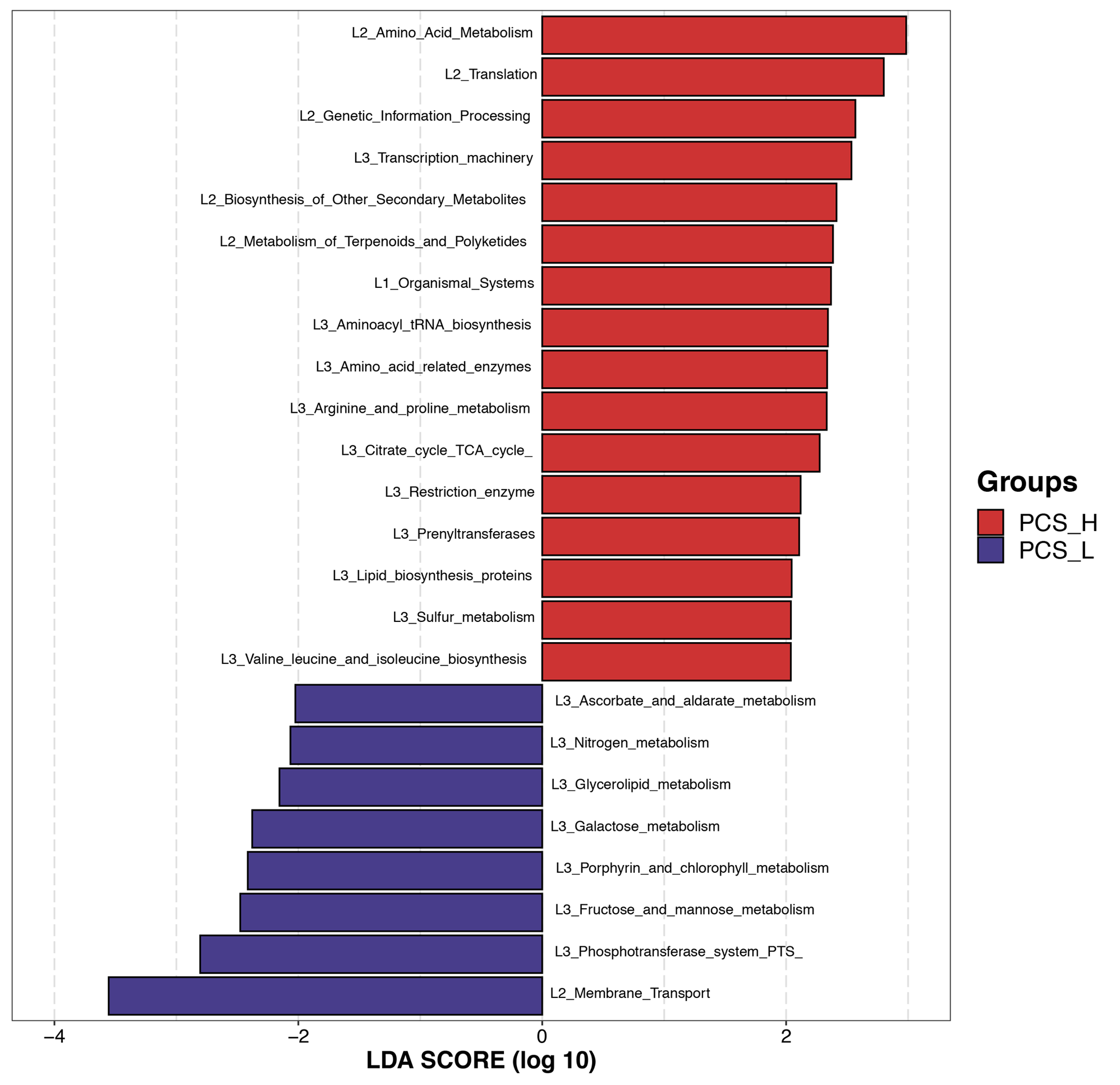

Supplement: Supplementary file 1 [file DataSheet_1.zip › All supplementary Figure and Data/s5 169*235.tif]

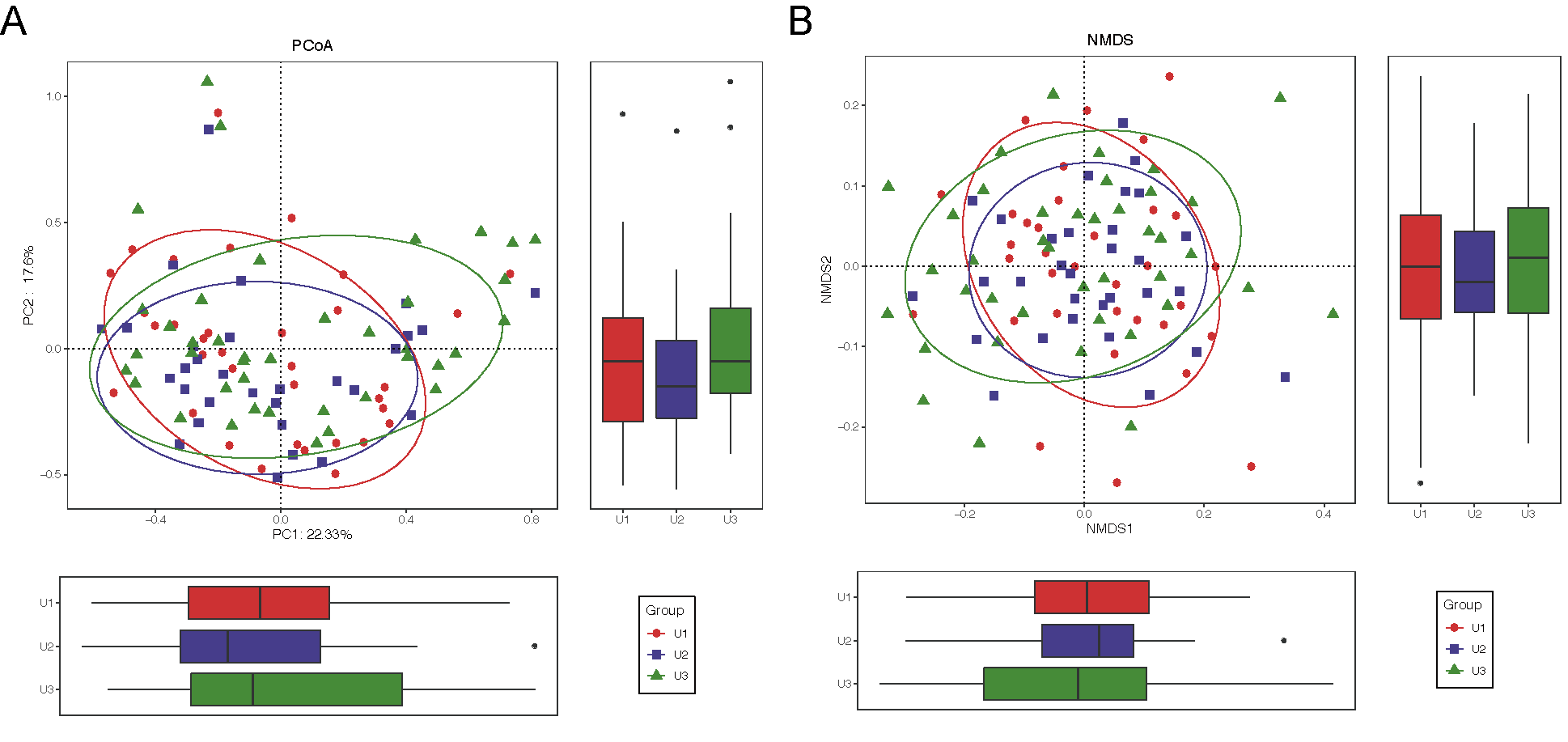

Supplement: Supplementary file 1 [file DataSheet_1.zip › All supplementary Figure and Data/s6 169*235.tif]
